# Telomere length is greater in ALS than in controls: a whole genome sequencing study

**DOI:** 10.1080/21678421.2019.1586951

**Published:** 2019-04-01

**Authors:** Ahmad Al Khleifat, Alfredo Iacoangeli, Aleksey Shatunov, Ton Fang, William Sproviero, Ashley R. Jones, Sarah Opie-Martin, Karen E. Morrison, Pamela J. Shaw, Christopher E. Shaw, John F. Powell, Richard Dobson, Steven J. Newhouse, Ammar Al-Chalabi

**Affiliations:** 1 Department of Basic and Clinical Neuroscience, King’s College London, Maurice Wohl Clinical Neuroscience Institute, London, UK;; 2 Department of Biostatistics and Health Informatics, King’s College London, London, UK;; 3 Faculty of Medicine, University of Southampton, University Hospital Southampton NHS Foundation Trust, Southampton, UK;; 4 Sheffield Institute for Translational Neuroscience, University of Sheffield, Sheffield, UK;; 5 King’s College Hospital, London, UK;; 6 Psychology and Neuroscience, United Kingdom Dementia Research Institute, Maurice Wohl Clinical Neuroscience Institute, Institute of Psychiatry, King’s College London, London, UK, and;; 7 Farr Institute of Health Informatics Research, UCL Institute of Health Informatics, University College London, London, UK

**Keywords:** *ALS*, *telomere*, *next-generation sequencing*, *whole genome sequencing*, *bioinformatics*, *variant calling*, *structural variants*

## Abstract

*Background:* Amyotrophic lateral sclerosis is a neurodegenerative disease of motor neurons resulting in progressive paralysis and death, typically within 3–5 years. Although the heritability of ALS is about 60%, only about 11% is explained by common gene variants, suggesting that other forms of genetic variation are important. Telomeres maintain DNA integrity during cellular replication and shorten naturally with age. Gender and age are risk factors for ALS and also associated with telomere length. We therefore investigated telomere length in ALS. *Methods:* We estimated telomere length by applying a bioinformatics analysis to whole genome sequence data of leukocyte-derived DNA from people with ALS and age and gender-matched matched controls in a UK population. We tested the association of telomere length with ALS and ALS survival. *Results:* There were 1241 people with ALS and 335 controls. The median age for ALS was 62.5 years and for controls, 60.1 years, with a male–female ratio of 62:38. Accounting for age and sex, there was a 9% increase of telomere length in ALS compared to matched controls. Those with longer telomeres had a 16% increase in median survival. Of nine SNPs associated with telomere length, two were also associated with ALS: rs8105767 near the *ZNF208* gene (*p* = 1.29 × 10^−4^) and rs6772228 (*p* = 0.001), which is in an intron for the *PXK* gene. *Conclusions:* Longer telomeres in leukocyte-derived DNA are associated with ALS, and with increased survival in those with ALS.

## Introduction

Amyotrophic lateral sclerosis is a neurodegenerative disease of motor neurons leading to progressive muscle weakness and death through neuromuscular respiratory failure (1). Although the heritability of ALS is about 60% (2), the heritability explained by common gene variants is only about 11% (3) suggesting that other forms of genetic variation play an important role.

Telomeres are repeated DNA sequences located at the ends of chromosomes and exist to maintain DNA integrity during cellular replication; chromosome ends tend to shorten with replication, and the repeat region protects against the loss of important gene sequences because loss of repeats can be tolerated (4). As such, telomeres shorten naturally with age as repeats are lost during replication cycles (5). Natural variation in telomere length exists in the population, with women on average having longer telomeres than men (6); shorter telomeres are associated with an increased risk of cancer (7).

A major risk factor for ALS is age (8) and ALS is also more common in men than women (9): both age and sex are related to telomere length. Furthermore, there are some similarities between ALS and cancer (10), such as evidence for a multistep process in pathogenesis (11,12). We therefore investigated telomere length in ALS.

## Materials and methods

### Whole-genome sequencing

Samples were from multiple centers across the UK contributing to the international Project MinE whole genome sequencing initiative (13).

DNA was isolated from venous blood using standard methods. The DNA concentrations were set at 100 ng/uL as measured by a fluorimeter with the PicoGreen^®^ dsDNA (Thermo Scientific, Waltham, MA) quantitation assay. DNA integrity was assessed using gel electrophoresis. All samples were sequenced using Illumina’s FastTrack services (Illumina, San Diego, CA) on the Illumina HiSeq 2000 platform (14). Sequencing was 100 bp paired-end performed using polymerase chain reaction (PCR)-free library preparations and yielded ∼40x coverage across each sample. Binary sequence alignment/map formats (BAM) were generated for each individual.

### Determination of telomere length

TelSeq (15) was used to quantify telomere length using data from whole genome sequences. Telomere lengths were estimated from reads, defined as repeats of more than seven TTAGGG motifs.

### Assessment of nine loci affecting mean telomere length and their association with ALS

We selected nine SNPs, reported in multiple genome-wide association studies (GWAS) as associated with mean telomere length in European-derived populations. The selected SNPs were rs6772228-*PXK* (16), rs9419958-*OBFC1* (17), rs9420907-*OBFC1* (18), rs4387287- *OBFC1* (10), rs3027234-*CTC1* (17), rs8105767-*ZNF208* (18), rs412658-*ZNF676* (17), rs6028466-*DHX35* (17), and rs755017-*ZBTB46* (17).

### Statistical analysis

The effects of telomere length on ALS were tested using a generalized linear regression model, which included total telomere length, age and sex, to predict disease affected status. To assess the model, Pearson’s chi-squared test was used.

Because telomere length correlates with age, we performed an additional test to examine the possibility that survival bias could affect the results. To do this, we also performed the analysis restricted to the subgroup of people with ALS onset below the median cohort age (62 years). Although such an analysis would halve our sample and therefore greatly reduce statistical power, the direction of effect should be observable.

To evaluate SNP effects on telomere length we calculated Nagelkerke’s *R*^2^ from the results of a generalized linear model using the value of telomere length, age, gender, and nine SNPs selected for having been previously shown to associate with telomere length.

To assess the effect of covariates on telomere length affecting survival, we used Cox regression, controlling for age, gender, and site of disease onset (bulbar or spinal).

To assess the association of genes with ALS we used the SNP-set sequence kernel association test (SKAT) (19), which is a test for association between a set of rare and common variants and continuous/dichotomous phenotypes using kernel machine methods.

Statistical tests were performed using IBM SPSS Statistics version 24.0 (SPSS Inc., Armonk, NY, USA) (20), RStudio, R Foundation for Statistical Computing version 3.4.1 (R Development Core Team, Vienna, Austria) (21).

### Ethical approval

Informed consent was obtained from all volunteers included in this project. Generation of whole genome sequences was approved by the Trent Research Ethics Committee 08/H0405/60.

## Results

There were 1241 people with apparently sporadic ALS and 335 controls. The median age for people with ALS was 62.5 years and for controls, 60.1 years, with a male–female ratio of 62:38 (Table 1).

**Table 1:** Demographics of the UK sample.

	ALS	Controls
Total *n*	1241	335
Male:Female ratio	766:475 (62% male)	124:211 (37% male)
Mean age	62.9 (SD 11.08)	60.1 (SD 11.47)

**Figure 1.**
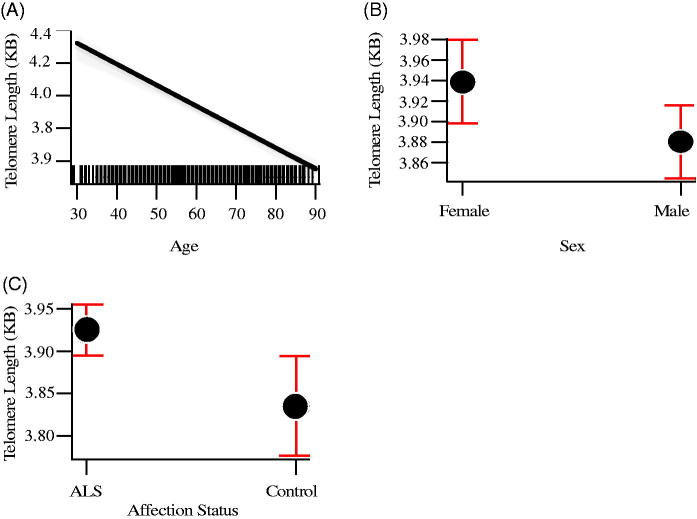
Mean telomere length by age (A), sex (B), and disease status (C). Red bars indicate 95% confidence intervals.

The mean telomere length in people with ALS was 3.95 kb, and in controls, 3.80 kb, not taking into account gender or age (Figure 1). Generalized linear regression accounting for these covariates showed a mean 9% (95% CI 3%, 15%) increase of telomere length in people with ALS compared to age and gender-matched controls (*p* = 0.008). In the analysis exploring survival bias as an explanation for our results, in which we restricted testing to those younger than the median age, the same direction of effect was observed, although as expected, because of the greatly reduced sample size, this did not reach statistical significance (*p* = 0.08). Covariate analysis showed that females (*p* = 0.03) and younger people (*p* = 2 × 10^−16^) had on average longer telomeres (Table 2), confirming the results of earlier studies that telomere length reduces with age and females have on average longer telomeres.

**Table 2:** Telomere length comparison between people with ALS and healthy controls using a generalized linear model.

	Estimate (%)	SE of estimate (%)	*p* Value
Age (per year)	−1	0.1	2 × 10^−16^
Gender (male *vs.* female)	−5	2	0.03
Case-control status (controls *vs.* cases)	−9	3	0.008

There was no association between telomere length and site of disease onset (*p* = 0.7), or with *C9orf72* expansion status (*p* = 0.24). 

Cox regression analysis showed that in the ALS group, those with longer telomeres had a 16% increase in median survival (hazard ratio 0.81 (95% CI 0.72–0.91), *p* = 0.001).

The generalized linear regression model showed that of the nine SNPs associated with telomere length, two were also associated with ALS: rs8105767 near the *ZNF208* gene (*p* = 1.29 × 10^−4^, MAF = 0.03) and rs6772228, which is in an intron for the *PXK* gene (*p* = 0.001, MAF = 0.03; Table 3), but the SKAT test did not show an association of overall variant burden in these genes with ALS after correction for multiple testing (*ZNF208*, *p* = 0.81 and *PXK*, *p* = 0.03). Nagelkerke’s *R*^2^ test showed that the nine selected SNPs contributed 3% to the variance in total telomere length.

**Table 3:** Assessment of telomere-associated SNPs.

SNP	Risk genotype	Beta	SE	*p* Value
**rs6772228**	**A/T**	**0.83**	**0.37**	**0.001**
**rs6772228**	**A/A**	**0.33**	**0.37**	**0.001**
rs9419958	T/T	0.61	0.56	0.036
rs9419958	C/T	0.59	0.54	0.031
rs9420907	C/C	0.59	0.53	0.021
rs9420907	A/A	0.6	0.53	0.024
rs4387287	A/A	0.71	0.16	0.014
rs4387287	A/C	0.63	0.07	0.067
rs3027234	G/G	0.62	0.07	0.009
rs3027234	A/G	0.63	0.07	0.011
**rs8105767**	**G/A**	**0.66**	**0.08**	**1.29 × 10^−4^**
**rs8105767**	**G/G**	**0.91**	**0.07**	**4.89 × 10^−4^**
rs412658	C/C	0.73	0.07	0.008
rs412658	C/T	0.69	0.06	0.058
rs6028466	G/G	0.36	0.22	0.087
rs6028466	A/G	0.37	0.22	0.088
rs755017	A/A	0.45	0.63	0.054
rs755017	A/G	0.45	0.63	0.052

Investigation of causal effect of telomere length on ALS by using nine SNPs identified by telomere length GWAS. SNPs rs8105767 (*PXK*) and rs6772228 (*ZNF208*) were associated with ALS.

SNPs highlighted in bold show association with ALS.

## Discussion

We have shown that longer telomeres are associated with ALS and with longer survival in ALS. In keeping with previous studies, we found that mean telomere length was longer in females and shortened with increasing age. Of a panel of nine SNPs known to be associated with telomere length, two showed association with ALS, one in *ZNF208*, and the other in *PXK*.

Although both these SNPs, rs6772228 and rs8105767, are known to be associated with telomere length, no association with ALS was seen in a previous large genome-wide association study (22), suggesting that either there is a population-specific effect, or that the telomere length itself is driving the association, and other factors that influence it have a larger effect than these SNPs. Another possibility is that the difference in results is because the analysis performed was different, as we have tested genotypic association, whereas the genome-wide association study used linear mixed modeling of alleles.

Telomeres have largely been investigated for their roles in cancer and aging, shorter telomeres being associated with disease pathology and death. Surprisingly, telomere elongation is also seen in about 15% of cancers, such as adenocarcinoma of the lung and pancreas (23), and in general, cancers with long telomeres are resistant to therapy and carry a poor prognosis (24). Telomere elongation phenomena are well documented but far less well understood than telomere shortening phenomena (24–28).

A study of telomere length in ALS brains found a trend to longer telomeres in glial cells (29) consistent with our results, but is in contrast to an earlier small study of 50 people with ALS and 50 controls, finding that shorter telomeres are associated with ALS (30).

Our study has some strengths and weaknesses. Although ALS is a disease of the central nervous system, our telomere data are derived from leukocyte DNA, since our DNA source was whole blood. The relationship between leukocyte telomeres, which can be expected to shorten with age as leukocytes undergo mitosis, and telomeres in neurons, which are post-mitotic, is not clear (31), but glial and other cells that do undergo mitosis are probably involved in ALS pathogenesis, and provide a possible mechanism. Furthermore, we did not directly measure telomere length using Southern blotting, but estimated it using whole genome sequence data. However, our findings have the advantage of a large sample size of more than 1200 cases, compared with previous reports of 50 or fewer. Furthermore, our examined cohort is more homogeneous in genetic background, and the sequencing technology used was the same across the entire cohort. However, one limitation of our method is that we cannot draw firm conclusions about the exact length of a telomere. The method we have used, TelSeq, correlates with results from Southern blotting (32), and Q-PCR (33) and is in widespread use (31,34). Nevertheless, different sequencing technologies will generate different telomere length estimates because of differences in library preparation and platform (35,36). To overcome this potential weakness, we have used the same industry-leading sequencing platform for all samples, as well as designing the study to minimize batch effects by having cases and controls sharing the same sequencing plate.

We found that longer telomeres were associated both with ALS and with increased survival in ALS. It is possible that telomere length does not associate with ALS risk but only with survival, and that our cohort was biased in such a way that those with longer survival were more likely to be genotyped. In that case, we would also observe an apparent association with risk, but the driver would be the actual association with increased survival. While this possibility cannot be completely excluded, the cohort tested was an incident cohort, collected from a population rather than a specialist clinic, reducing the likelihood of this explanation. Furthermore, we assessed survival bias by testing the relationship between telomere length and ALS in the younger half of the sample. We found the direction of association of longer telomeres with ALS was still present, although as expected, the statistical power was reduced due to the smaller number of young controls (<175). Replicating these findings in a bigger cohort such as the entire Project MinE sample is an important future step.

There are multiple methods available for telomere length analysis, including terminal restriction fragmentation, quantitative fluorescence *in situ* Hybridization (Q-FISH) (37), PCR-based techniques and southern blotting. These techniques have the disadvantage of lengthy protocols and limitations, such as the requirement that DNA is extracted from fresh blood, or that chromosomes are individually stained, which is a time-consuming process (35,38–41). Differences in applying these techniques between laboratories can create measurement differences (41). Thus, for large scale analyses, whole genome sequence data that can be processed using a standard bioinformatics pipeline can standardize measurements and overcome many of these issues (42). We have shown that measuring telomere length in a UK cohort is feasible using a bioinformatics tool, such as TelSeq, and that this is fast and cost-effective. Estimating the telomere length with TelSeq on a single 40x whole genome sequence takes about 90 min using four threads on a midrange computer, which would translate to about 100 days for our entire dataset. Since high-performance computing access is now straightforward, and multiple computers are able to run the same analysis in parallel, the analysis time can easily be shortened significantly.

In this large study of telomere length and ALS, we have shown that longer telomeres in leukocytes are associated with ALS, and with increased survival in those with ALS.

## References

[1] Brown RH, Al-Chalabi A Amyotrophic lateral sclerosis. N Engl J Med. 2017;377:162–72. 28700839 10.1056/NEJMra1603471

[2] Al-Chalabi A, Fang F, Hanby MF, Leigh PN, Shaw CE, Ye W, et al. An estimate of amyotrophic lateral sclerosis heritability using twin data. J Neurol Neurosurg Psychiatry. 2010;81:1324–6. 20861059 10.1136/jnnp.2010.207464 PMC2988617

[3] McLaughlin RL, Vajda A, Hardiman O Heritability of amyotrophic lateral sclerosis: insights from disparate numbers. JAMA Neurol. 2015;72:857–8. 26029874 10.1001/jamaneurol.2014.4049

[4] Muzumdar R, Atzmon G. Telomere Length and Aging. Bibo Li, IntechOpen. 2012 DOI:10.5772/53227 Available from: https://www.intechopen.com/books/reviews-on-selected-topics-of-telomere-biology/telomere-length-and-aging

[5] Kong CM, Lee XW, Wang XY Telomere shortening in human diseases. Febs J. 2013;280:3180–93. 23647631 10.1111/febs.12326

[6] Gardner M, Bann D, Wiley L, Cooper R, Hardy R, Nitsch D, et al. Gender and telomere length: systematic review and meta-analysis. Exp Gerontol. 2014;51:15–27. 24365661 10.1016/j.exger.2013.12.004 PMC4523138

[7] Xu LF, Li S, Stohr BA The role of telomere biology in cancer. Annu Rev Pathol. 2013;8:49–78. 22934675 10.1146/annurev-pathol-020712-164030

[8] Al-Chalabi A ,Hardiman O The epidemiology of ALS: a conspiracy of genes, environment and time. Nat Rev Neurol. 2013;9:617–28. 24126629 10.1038/nrneurol.2013.203

[9] McCombe PA, Henderson RD Effects of gender in amyotrophic lateral sclerosis. Gend Med. 2010;7:557–70. 21195356 10.1016/j.genm.2010.11.010

[10] Levy D, Neuhausen SL, Hunt SC, Kimura M, Hwang SJ, Chen W, et al. Genome-wide association identifies OBFC1 as a locus involved in human leukocyte telomere biology. Proc Natl Acad Sci. 2010;107:9293–8. 20421499 10.1073/pnas.0911494107 PMC2889047

[11] Al-Chalabi A, Calvo A, Chio A, Colville S, Ellis CM, Hardiman O, et al. Analysis of amyotrophic lateral sclerosis as a multistep process: a population-based modelling study. Lancet Neurol. 2014;13:1108–13. 25300936 10.1016/S1474-4422(14)70219-4 PMC4197338

[12] Chiò A, Mazzini L, D’Alfonso S, Corrado L, Canosa A, Moglia C, et al. The multistep hypothesis of ALS revisited: the role of genetic mutations. Neurology. 2018;91:e635–e42. 30045958 10.1212/WNL.0000000000005996 PMC6105040

[13] Van Rheenen W, Pulit SL, Dekker AM, Al Khleifat A, Brands WJ, Iacoangeli A, et al. Project MinE: study design and pilot analyses of a large-scale whole-genome sequencing study in amyotrophic lateral sclerosis. Eur J Hum Genet. 2018;26:1537–46. 29955173 10.1038/s41431-018-0177-4 PMC6138692

[14] Illumina HiSeq™ 2000 sequencing system. *Specification sheet: illumina^®^ sequencing*. 2010:1–4.https://www.illumina.com/Documents/products/datasheets/datasheet_hiseq2500.pdf

[15] Ding Z, Mangino M, Aviv A, Durbin R, UK10K Consortium, Tim Spector. et al. Estimating telomere length from whole genome sequence data. Nucleic Acids Res. 2014;42:e75. 24609383 10.1093/nar/gku181 PMC4027178

[16] Pooley KA, Bojesen SE, Weischer M, Nielsen SF, Thompson D, Amin Al Olama A, et al. A genome-wide association scan (GWAS) for mean telomere length within the COGS project: identified loci show little association with hormone-related cancer risk. Hum Mol Genet. 2013;22:5056–64. 23900074 10.1093/hmg/ddt355 PMC3836481

[17] Mangino M, Hwang SJ, Spector TD, Hunt SC, Kimura M, Fitzpatrick AL, et al. Genome-wide meta-analysis points to CTC1 and ZNf676 as genes regulating telomere homeostasis in humans. Hum Mol Genet. 2012;21:5385–94. 23001564 10.1093/hmg/dds382 PMC3510758

[18] Codd V, Nelson CP, Albrecht E, Mangino M, Deelen J, Buxton JL, et al. Identification of seven loci affecting mean telomere length and their association with disease. Nat Genet. 2013;45:422–7, 427e1–2. 23535734 10.1038/ng.2528 PMC4006270

[19] Ionita-Laza I, Lee S, Makarov V, Buxbaum JD, Lin X Sequence kernel association tests for the combined effect of rare and common variants. Am J Hum Genet. 2013;92:841–53. 23684009 10.1016/j.ajhg.2013.04.015 PMC3675243

[20] IBM Corp. Released 2016. IBM SPSS Statistics for Macintosh, Version 24.0. Armonk, NY: IBM Corp. URL https://www.ibm.com/uk-en/products/spss-statistics

[21] RStudio Team. RStudio: Integrated Development for R. RStudio, Inc., Boston, MA. 2015. URL https://www.rstudio.com/.

[22] van Rheenen W, Shatunov A, Dekker AM, McLaughlin RL, Diekstra FP, Pulit SL, et al. Genome-wide association analyses identify new risk variants and the genetic architecture of amyotrophic lateral sclerosis. Nat Genet. 2016;48:1043–8. 27455348 10.1038/ng.3622 PMC5556360

[23] Min J, Wright WE, Shay JW Alternative lengthening of telomeres can be maintained by preferential elongation of lagging strands. Nucleic Acids Res. 2017;45:2615–28. 28082393 10.1093/nar/gkw1295 PMC5389697

[24] Haycock PC, Burgess S, Nounu A, Zheng J, Okoli GN, Bowden J, et al. Association between telomere length and risk of cancer and non-neoplastic diseases a Mendelian randomization study. JAMA Oncol. 2017;3:636–51. 28241208 10.1001/jamaoncol.2016.5945 PMC5638008

[25] Bryan TM, Englezou A, Gupta J, Bacchetti S, Reddel RR Telomere elongation in immortal human cells without detectable telomerase activity. Embo J. 1995;14:4240–8. 7556065 10.1002/j.1460-2075.1995.tb00098.x PMC394507

[26] Blackburn EH, Greider CW, Szostak JW Telomeres and telomerase: the path from maize, Tetrahymena and yeast to human cancer and aging. Nat Med. 2006;12:1133–8. 17024208 10.1038/nm1006-1133

[27] Cesare AJ, Reddel RR Alternative lengthening of telomeres: models, mechanisms and implications. Nat Rev Genet. 2010;11:319–30. 20351727 10.1038/nrg2763

[28] Arora R, Azzalin CM Telomere elongation chooses TERRA ALTernatives. RNA Biol. 2015;12:938–41. 26158306 10.1080/15476286.2015.1065374 PMC4615670

[29] Linkus B, Wiesner D, Meßner M, Karabatsiakis A, Scheffold A, Rudolph KL, et al. Telomere shortening leads to earlier age of onset in ALS mice. Aging (Albany NY). 2016;8:382–93. 26978042 10.18632/aging.100904 PMC4789589

[30] De Felice B, Annunziata A, Fiorentino G, Manfellotto F, D’Alessandro R, Marino R, et al. Telomerase expression in amyotrophic lateral sclerosis (ALS) patients. J Hum Genet. 2014;59:555–61. 25142509 10.1038/jhg.2014.72

[31] Barthel FP, Wei W, Tang M, Martinez-Ledesma E, Hu X, Amin SB, et al. Systematic analysis of telomere length and somatic alterations in 31 cancer types. Nat Genet. 2017;49:349–57. 28135248 10.1038/ng.3781 PMC5571729

[32] Ding Z, Mangino M, Aviv A, Spector T, Durbin R Estimating telomere length from whole genome sequence data. Nucleic Acids Res. 2014;42:e75. 24609383 10.1093/nar/gku181 PMC4027178

[33] Cook DE, Zdraljevic S, Tanny RE, Seo B, Riccardi DD, Noble LM, et al. The genetic basis of natural variation in *Caenorhabditis elegans* telomere length. Genetics. 2016;204:371–83. 27449056 10.1534/genetics.116.191148 PMC5012401

[34] Cai N, Chang S, Li Y, Li Q, Hu J, Liang J, et al. Molecular signatures of major depression. Curr Biol. 2015;25:1146–56. 25913401 10.1016/j.cub.2015.03.008 PMC4425463

[35] Ma TS Applications and limitations of polymerase chain reaction amplification. Chest. 1995;108:1393–404. 7587447 10.1378/chest.108.5.1393

[36] Aviv A, Hunt SC, Lin J, Cao X, Kimura M, Blackburn E, et al. Impartial comparative analysis of measurement of leukocyte telomere length/DNA content by Southern blots and qPCR. Nucleic Acids Res. 2011;39:e134. 21824912 10.1093/nar/gkr634 PMC3203599

[37] Baerlocher GM, Lansdorp PM Telomere length measurements in leukocyte subsets by automated multicolor flow-FISH. Cytometry A. 2003;55:1–6. 12938182 10.1002/cyto.a.10064

[38] Eastmond DA, Schuler M, Rupa DS Advantages and limitations of using fluorescence in situ hybridization for the detection of aneuploidy in interphase human cells. Mutat Res Lett. 1995;348:153–62. 10.1016/0165-7992(95)90003-9 8544867

[39] Baerlocher GM, Lansdorp PM Telomere length measurements using fluorescence in situ hybridization and flow cytometry. Methods Cell Biol. 2004;75:719–50. 15603450 10.1016/s0091-679x(04)75031-1

[40] Baerlocher GM, Mak J, Tien T, Lansdorp PM Telomere length measurement by fluorescence in situ hybridization and flow cytometry: tips and pitfalls. Cytometry. 2002;47:89–99. 11813198 10.1002/cyto.10053

[41] Aviv A, Valdes AM, Spector TD Human telomere biology: pitfalls of moving from the laboratory to epidemiology. Int J Epidemiol. 2006;35:1424–9. 16997848 10.1093/ije/dyl169

[42] Barrett JH, Iles MM, Dunning AM, Pooley KA Telomere length and common disease: study design and analytical challenges. Hum Genet. 2015;134:679–89. 25986438 10.1007/s00439-015-1563-4 PMC4460268

